# Twenty years of Primafamed Network in Africa: Looking back at the future

**DOI:** 10.4102/phcfm.v9i1.1603

**Published:** 2017-09-21

**Authors:** Jan De Maeseneer

**Affiliations:** 1 International Centre for Family Medicine and Primary Health Care, Ghent University, WHO Collaborating Centre on Primary Health Care, Belgium

In September 1997 in Durban (South Africa), a workshop was organised with the eight South African departments and the four Flemish departments of Family Medicine and Primary Care in order to explore opportunities for cooperation. In the ‘Durban Declaration’,^1^ the eight South African departments agreed to establish a consortium for cooperation to develop a common vision for vocational training in family medicine. The Family Medicine Educational Consortium (FaMEC) organised regularly inter-university meetings to develop a common curriculum. In 2003, FaMEC obtained a grant from the Flemish Inter-university Council (VLIRUOS) for the project ‘Optimisation of the Vocational Medical Training in Family Medicine/Primary Health Care in South Africa: A contribution to the realisation of health for all’. Prof. J. Hugo (University of Pretoria) acted as the first coordinator and stimulated development of a ‘Train the Trainer’ programme. Practical training was organised in ‘training complexes’, consisting of community health centres with the cooperating ‘clinics’ and ‘district hospitals’. In 2006, there was an extension of the programme, involving institutions like Aga Khan University (Tanzania), Moi University (Eldoret, Kenya), University of Goma (Democratic Republic of Congo), National University of Rwanda (Kigali-Huye), Makerere University (Kampala) and Mbarara University of Science and Technology, both in Uganda. The financing by the Belgian Developmental Cooperation Agency and a ‘South-South-Strategy’ with mutual information sharing contributed to appropriate educational models, taking into account the different contexts and health systems.

In 2008, for the first time the name of ‘Primafamed Network’ was used (Primary Care/Family Medicine Educational Network), when the project acquired financing by the ACP-EU Cooperation programme in Higher Education (EDULINK). The focus was broadened to strengthening training capacity of academic departments of family medicine both in undergraduate and postgraduate training. Institutions of Sudan, Ghana and Nigeria joined the network. An important landmark in the development of the Primafamed Network was the launch of the ‘African Journal for Primary Health Care and Family Medicine’ in 2008, with a small seed grant from Belgian Development Aid, at the first ‘Primafamed Workshop’ in Kampala (Uganda), a gathering of more than 100 stakeholders in Family Medicine and Primary Health Care from sub-Saharan countries.

Apart from the focus on education and training, increasingly research and publications underpinned the basic principles of Family Medicine in Africa. Mash used a Delphi consensus procedure and described the basic principles of Family Medicine in Africa.^2^

From June 2009, the South–South cooperation was formalised in the project ‘Strengthening Developmental Capacity for Family Medicine Training in Africa: The South African Family Medicine Twinning Project’. Each of the departments of Family Medicine in South Africa started a ‘Twinning’ in order to train family physicians in countries and regions where there was not a medical faculty or even a university.

A successful ‘Africanisation’ of the Primafamed Network took place when the Department of Family Medicine and Primary Healthcare of Stellenbosch University made a successful application for a grant from the European Union for a project aiming at scaling up dramatically the number of physicians in primary care in South Africa. In 2012, the need for scaling up capacity was echoed by the ‘Statement of the Primafamed Network, Victoria Falls, Zimbabwe: Scaling up family medicine and primary health care in Africa’.^3^ This document made a plea for increasing the percentage of medical graduates that should be trained in family medicine.

The Primafamed Network could take advantage of ‘best practices’, for example the 2-year training programme in family medicine in the community implemented by the Faculty of Medicine of Gezira University and the Ministry of Health in Sudan. In this programme, starting from the needs of the local population and supported by technology (E-Learning and Telemedicine), 207 candidates were trained directly in the community in 158 health centres, of which 84 had never been served by a doctor before.^4^

Flinkenflögel et al. assessed the impact of the Primafamed Project looking at the developmental progress at the level of the participating departments of family medicine and primary health care in the period 2008–2011. All departments made considerable progress, and the Strengths, Weaknesses, Opportunities, Threats analysis illustrated that support from local authorities for the departments is of utmost importance. Training of family physicians is possible, but is a slow process and South–South cooperation is an effective strategy.^5^

What is the future of the Primafamed Network nowadays in 2017? In recent years, the willingness to fund projects like Primafamed by international donors and agencies has decreased. Fortunately, the Primafamed Network could take advantage of the ‘Medical Education Performance Initiative’ (MEPI), but the new Trump administration seems not to be interested in supporting Africa. Moreover, many resources still go to vertical disease-oriented programmes, although the WHA62.12 resolution ‘Primary care, including health systems strengthening’ emphasised the need to integrate and develop disease-oriented programmes in the framework of comprehensive primary health care.^6^

In order to increase the sustainability of the Primafamed Network, different actions are needed: increasing the ownership by African institutions, strengthening the links with other organisations in the field of primary care and family medicine, for example WONCA Africa, and broadening the international support. Apart from the Department of Family Medicine and Primary Health Care of Ghent University and the three other Flemish departments, groups from University of Amsterdam (The Netherlands) and the University of Aarhus (Denmark) and from the General Practice Development and Research Centre, Peking University Health Science Centre (China), have also been supportive.

But it will be very important that the Primafamed Network is more ‘visible’ in Africa: a recent publication on ‘Current status of family medicine faculty development in sub-Saharan Africa’^7^ does not mention the Primafamed Network at all.

The renewed interest of WHO in strengthening primary health care could be an important asset for the Primafamed Network. Demonstrating the evidence that the development of healthy, equitable and sustainable societies needs strong primary health care to build the social cohesion and solidarity that will shape our future should inspire politicians to take appropriate decisions in developing health systems.^8^
